# Multiple Masses on the Tongue of a Patient with Generalized Mucocutaneous Lesions

**DOI:** 10.1371/journal.pmed.0050212

**Published:** 2008-11-11

**Authors:** Luca Pastore, Maria Luisa Fiorella, Raffaele Fiorella, Lorenzo Lo Muzio

## Abstract

Luca Pastore and colleagues discuss the differential diagnosis and management of a 62-year-old man who presented with a 6-cm fungating mass on the dorsum of the tongue.

## PRESENTATION OF CASE

In May 2007, a 62-year-old man presented with a 6-cm fungating mass on the dorsum of the tongue (Figure 1), which had lasted for an unknown period of time. The lesion had a roughened and irregular surface, with areas of white keratosis admixed with areas of necrosis. It felt indurated on palpation. Two additional smaller exophytic lesions were observed on the dorsum of the tongue: one adjacent to the main mass and the other on the apex. Both showed surface nodularity and minimal surface keratin production. The surrounding mucosa demonstrated hyperkeratosis and a pebbled surface. Interlacing white keratotic striae were observed on the right buccal mucosa (Figure 2), while the mucosa of the left cheek had an erosive area bordered by fine, white radiating striae (not shown in figure).

**Figure 1:**
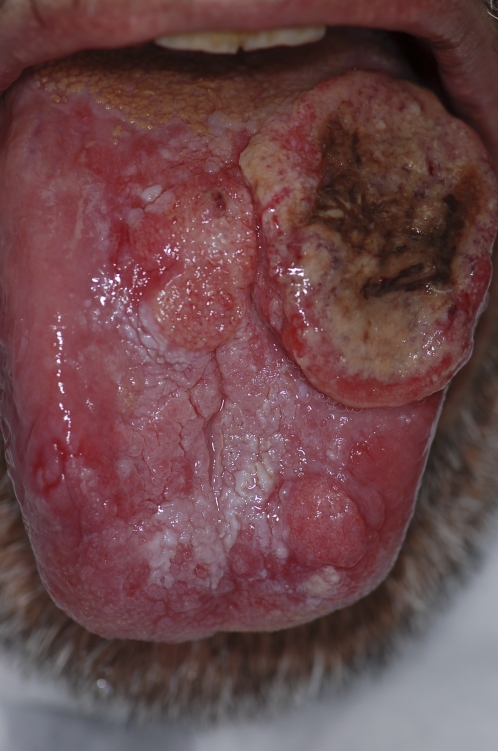
The Dorsum of the Patient's Tongue break;On biopsy, the three exophytic masses turned out to be oral carcinomas, while the surrounding hyperkeratotic area showed histologic features of oral lichen planus.

**Figure 2:**
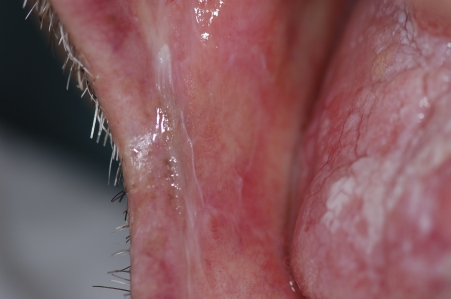
Interlacing White Striae on the Right Buccal Mucosa, Consistent with the Diagnosis of Oral Lichen Planus

A skin examination revealed polygonal, flat-topped papules covered with a fine network of white lines on the legs (Figure 3). Furthermore, the patient had an erosive area on his glans penis (Figure 4).

**Figure 3:**
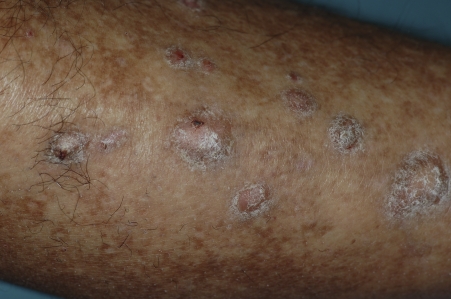
Polygonal, Flat-Topped Papules Covered with a Fine Network of White Lines on the Legs, Consistent with the Diagnosis of Lichen Planus

**Figure 4:**
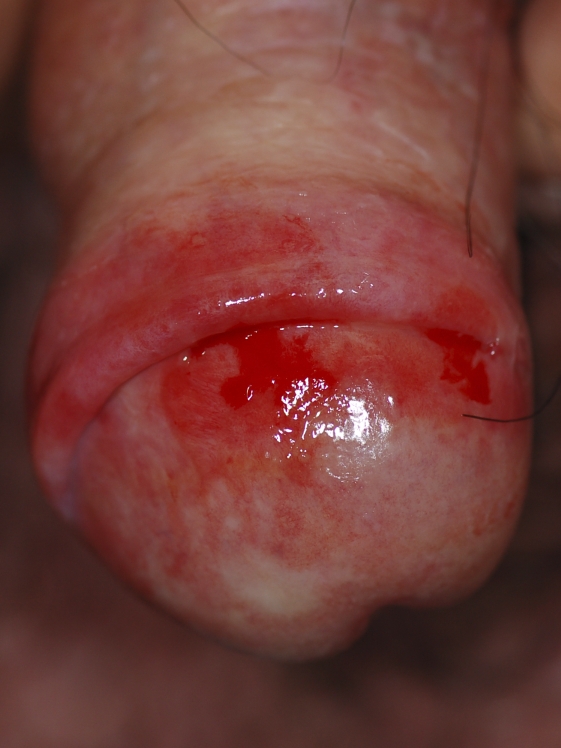
Erosive Area on the Glans Penis, Consistent with the Diagnosis of Lichen Planus

### What Clinical Diagnoses Were Considered?

The clinical appearance of the fungating mass immediately raised a high suspicion of malignancy, and a biopsy was promptly planned. For the two other exophytic lesions on the dorsum of the tongue, the differential diagnosis included squamous cell carcinoma, verrucous carcinoma, squamous papilloma, condyloma acuminatum, focal epithelial hyperplasia, and verruciform xanthoma.

The differential diagnosis of the hyperkeratotic surrounding area included plaque-like lichen planus, leukoplakia (thick or verruciform), proliferative verrucous leukoplakia, and lichenoid mucositis.

With regard to the buccal lesions, a clinical diagnosis of oral lichen planus (OLP) was relatively straightforward, given the presence of the bilateral interlacing white keratotic striae. Similarly, the skin lesions were highly suggestive of lichen planus, with the typical polygonal papules covered with a fine, lace-like network of white lines (so-called Wickham striae; in analogy, the interlacing white lines of reticular OLP are also referred to as Wickham striae by some authors).

The erosive area on the glans penis could be erosive lichen planus, pemphigus vulgaris, mucous membrane pemphigoid, plasma cell balanitis (or Zoon balanitis), or erythroplasia of Queyrat.

### Progress

On biopsy, the fungating mass on the dorsum of the tongue showed features of an invasive squamous cell carcinoma, while the contiguous exophytic lesion turned out to be a verrucous carcinoma; the mass on the apex of the tongue was a squamous cell carcinoma in situ. Histologic appearance of the hyperkeratotic surrounding area was consistent with OLP.

Biopsies of either buccal and cutaneous lesions were not performed; nonetheless, both were diagnosed as lichen planus on the basis of the striking clinical findings alone. A biopsy of the genital lesion was not considered a priority at that time; in accordance with the concomitant oral and skin involvement, a presumptive diagnosis of erosive lichen planus was made.

### Which Tests Would Now Be Helpful?

Once a biopsy-proven diagnosis of oral cancer is made, a staging workup is necessary in order to determine the extent of the primary tumor, the regional lymph node status, and the presence of distant metastasis. For this purpose, a comprehensive head and neck examination and imaging studies (computed tomography and/or magnetic resonance imaging; more recently, also positron emission tomography) are performed, and the cancer is staged according to the TNM (tumor, node, metastasis) system [1] (Tables 1 and 2). Depending on the clinical stage, location, and histologic findings of the primary cancer, general health status of the patient, and the patient's wishes, treatment of oral cancer is individually planned for each case.

**Table 1:**
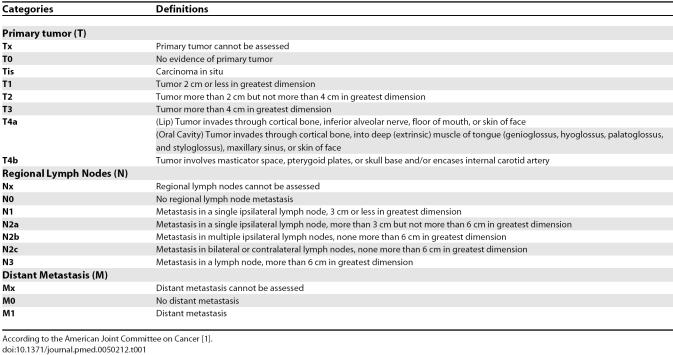
TNM Classification System for Oral Squamous Cell Carcinoma

**Table 2:**
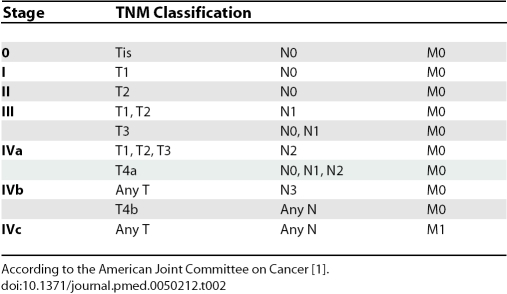
Stage Grouping

In this patient, total-body computed tomographic scans revealed multiple enlarged lymph nodes in the left laterocervical and submandibular regions, while there was no evidence of distant metastasis. On the basis of clinical and imaging data, the invasive tumor was staged as T4 N2 M0.

### Progress

The patient underwent surgery, with partial glossectomy and ipsilateral neck dissection. On histologic examination, the invasive cancer was identified as moderately differentiated squamous cell carcinoma that had widely invaded into muscles. Lymph node metastases were moderately differentiated squamous cell carcinomas as well, with no extracapsular extension. Based on additional data acquired from pathologic examination, the pathologic staging (pTNM) was T4a N2b M0 (stage IVa). Resection margins were free of tumor, and postoperative radiotherapy was given only on the neck.

The general health status of the patient rapidly worsened after surgery, and at a two-month follow-up evaluation a new mass was observed on the base of the tongue. On biopsy, this was diagnosed as a new squamous cell carcinoma, but a second surgical treatment was not planned, given the very poor health status of the patient. Combined radiotherapy and chemotherapy were prescribed, but the patient died two months later.

The family gave written consent for details of this case to be published.

## DISCUSSION

Oral cancer is a global health problem; worldwide, nearly 275,000 patients are annually estimated to have oral cancer, which represents about 3% of all malignancies in men and 2% in women [2]. The vast majority of oral cancers (approximately 94%) are squamous cell carcinomas that arise from the epithelium of the oral mucosa [3]. Survival of patients with oral cancer is directly related to the stage of disease at diagnosis; in fact, the five-year relative survival rate increased from 26.5% for oral cancers diagnosed at a distant stage to 81.8% for cases diagnosed at a localized stage [4]. Squamous cell carcinomas of the tongue seem to have lower rates of overall survival compared with cancers located in other oral cavity sites [5]. Unfortunately, only 33% of oral cancers are diagnosed at a localized stage, when the disease may be more easily and successfully treated [6]. This fact is responsible for a relatively poor overall five-year survival rate (59.1%), which has not significantly improved in the past several decades [6]. As a consequence, early detection is critical to reduce oral cancer mortality. In 9% to 25% of patients with oral cancer, additional synchronous or metachronous primary oral or pharynx carcinomas develop [3]; for these patients, the survival rates are worse.

Current therapeutic options for oral cancer include surgery, radiotherapy, and chemotherapy (each alone or in various combinations). In general, a wide surgical excision of the primary tumor remains the first line of treatment, even though stage I and stage II oral cancers could also be successfully treated with radiotherapy alone [7]. For stage III and stage IV oral cancers, surgery may be combined with radiotherapy and/or chemotherapy. A postoperative radiotherapy leads to better outcomes than radiotherapy administered preoperatively [8]. Indications for a postoperative radiation therapy include: positive surgical resection margins, bone or perineural invasion, multiple positive lymph nodes, or extracapsular lymph node invasion [9].

Chemotherapy can be given before surgery, after surgery and before radiotherapy, at the same time as radiotherapy, or alternating with radiotherapy. There is some evidence that postoperative concurrent radiotherapy and chemotherapy are more effective than postoperative radiotherapy alone in patients with advanced oral cancers [10], although this combined treatment may increase complications [11]. When there is clinical and/or imaging evidence of cervical lymph node metastasis, a neck dissection is required. Elective selective neck dissection may be performed in the node-negative neck, given the possibility of occult neck lymphadenopathy. Size and location of the primary lesion, thickness of the tumor, and invasion of neural, vascular, or lymphatic structures are factors used to evaluate the risk of occult cervical metastasis [12].

However, primary prevention and early diagnosis remain the cornerstones of strategies for reducing oral cancer mortality. With regard to primary prevention, public education efforts must be made to encourage people to avoid high-risk behaviors, with tobacco smoking and alcohol consumption being the major risk factors. As to early detection, the American Cancer Society recommends a periodic cancer-related check-up, which should include examination for oral cancers [13], and guidelines for visual inspection of the oral cavity were suggested [14]. However, a recent Cochrane review concluded that there is insufficient evidence to recommend screening of the general population for oral cancer using visual examination or adjunctive tools (toluidine blue, brush biopsy, fluorescence imaging) as an effective method for decreasing oral cancer mortality [15]. Nonetheless, the authors recommended regular screening by visual inspection applied by qualified health care providers for high-risk groups [15]. Patients with potentially malignant disorders of the oral mucosa have an increased risk of development of oral cancer; for these patients, close monitoring is recommended [16].

Lichen planus is an immune-mediated chronic inflammatory disease that commonly affects the epithelium of the oral mucosa with a spectrum of clinical conditions, including keratotic, atrophic, erosive, and ulcerative lesions; epithelia of other sites can be involved, namely the skin, the glans penis, the vaginal and vulvar mucosa, and the scalp [17]. A simultaneous involvement of more than one mucocutaneous site occurs in a relatively high percentage of patients with lichen planus [18].

A number of studies have indicated that OLP may be a precancerous lesion, with reported rates of malignant transformation that range from 0.4% to 5.6% [19]. Nevertheless, the precancer character of OLP remains highly controversial; the criticism is largely based on the vagueness of the criteria used in many studies for the diagnosis of OLP [19]. It has been argued that a transformation rate of even 1% would imply that nearly all oral cancers would develop from OLP, which is actually unlikely [20]. Moreover, applying strict diagnostic criteria, a recent study found that only oral lichenoid lesions and not OLP are associated with an increased risk of malignant transformation [19], although it is important to note that the concept of oral lichenoid lesion is somewhat controversial as well [18]; at any rate, the authors do not advise monitoring of patients with OLP [19]. In addition, at present there are no data to demonstrate the effectiveness of long-term follow-up of patients with OLP in reducing morbidity and mortality from oral cancer [21]. Therefore, to date we have no robust evidence to support the hypothesis of a malignant potential of OLP, and evidence to justify a continuous recall of patients with this oral ailment is lacking as well.

In spite of this, our report should alert physicians and dental practitioners that a potential of malignant transformation of OLP presumably exists, although its actual magnitude is yet to be established. Two other worrying facts should be taken into account: a study of 2,071 patients with cutaneous lichen planus, with no information about the oral status, reported no increase in risk of skin cancer, while the risk of oral cancer was 5.9-fold increased [22]; another study involving patients with OLP who subsequently had one oral carcinoma found that additional synchronous or metachronous mouth malignancies developed in 56% of participants [23].

**Key Learning Points**

- Oral cancer is a global health problem with a relatively poor prognosis, mainly due to late diagnosis; as a consequence, early detection is critical to reduce mortality.
- There is insufficient evidence to support the effectiveness of current screening methods in decreasing oral cancer mortality; however, close monitoring of patients with potentially malignant disorders of the oral mucosa is recommended.
- There is ongoing controversy as to the potentially malignant nature of oral lichen planus, and there is insufficient evidence to support the effectiveness of long-term monitoring of patients with oral lichen planus in reducing morbidity and mortality of oral cancer.
- This report should alert physicians and dental practitioners that a potential of malignant transformation of oral lichen planus presumably exists, although its actual magnitude is yet to be established.
- Well-powered prospective studies are needed to provide the highest levels of evidence for practice; in the meantime, long-term monitoring of patients with oral lichen planus should be advisable.

There is no doubt that further well-designed and well-powered long-term prospective studies, with validated diagnostic criteria for OLP, are needed to provide the highest levels of evidence for practice; in the meantime, long-term monitoring of patients with OLP should be advisable. The presence of generalized, mucocutaneous lesions could be a matter of greater concern.

## Abbreviations

OLP: oral lichen planus
TNM: tumor, node, metastasis
